# *Drosophila* CENP-A Mutations Cause a BubR1- Dependent Early Mitotic Delay without Normal Localization of Kinetochore Components

**DOI:** 10.1371/journal.pgen.0020110

**Published:** 2006-07-14

**Authors:** Michael D Blower, Tanya Daigle, Thom Kaufman, Gary H Karpen

**Affiliations:** 1 Department of Molecular and Cell Biology, University of California Berkeley, Berkeley, California, United States of America; 2 Department of Anesthesiology, University of Washington, Seattle, Washington, United States of America; 3 Department of Biology, Indiana University, Bloomington, Indiana, United States of America; 4 Department of Genome Biology, Lawrence Berkeley National Laboratory, Berkeley, California, United States of America; Stowers Institute for Medical Research, United States of America

## Abstract

The centromere/kinetochore complex plays an essential role in cell and organismal viability by ensuring chromosome movements during mitosis and meiosis. The kinetochore also mediates the spindle attachment checkpoint (SAC), which delays anaphase initiation until all chromosomes have achieved bipolar attachment of kinetochores to the mitotic spindle. CENP-A proteins are centromere-specific chromatin components that provide both a structural and a functional foundation for kinetochore formation. Here we show that cells in *Drosophila* embryos homozygous for null mutations in CENP-A (CID) display an early mitotic delay. This mitotic delay is not suppressed by inactivation of the DNA damage checkpoint and is unlikely to be the result of DNA damage. Surprisingly, mutation of the SAC component BUBR1 partially suppresses this mitotic delay. Furthermore, *cid* mutants retain an intact SAC response to spindle disruption despite the inability of many kinetochore proteins, including SAC components, to target to kinetochores. We propose that SAC components are able to monitor spindle assembly and inhibit cell cycle progression in the absence of sustained kinetochore localization.

## Synopsis

Normal inheritance of genetic traits from one cell or organismal generation to the next depends on accurate chromosome replication and segregation. Defective chromosome segregation is associated with birth defects and cancer. The centromere is a single site on the chromosome that is responsible for assembling the kinetochore, which mediates chromosome attachment to the microtubule spindle and all chromosome movements. In addition, the spindle assembly checkpoint (SAC) ensures normal inheritance by delaying entry into anaphase when chromosome–spindle attachments are defective. Previous studies suggested that SAC function required kinetochore localization of key components. This study shows that elimination of a centromere-specific histone (CID) results in an early mitotic delay. Although this delay occurs earlier than the established time of SAC function (at the metaphase–anaphase transition), it depends on the presence of an essential SAC protein (BUBR1). Furthermore, the CID-mediated early mitotic delay occurs in the absence of kinetochore formation or localization of key SAC proteins. These results suggest that the fidelity of kinetochore–microtubule attachment is also monitored early in mitosis, and in the absence of kinetochore formation and localization of SAC components.

## Introduction

Proper kinetochore assembly and function is essential for the faithful transmission of chromosomes during both mitosis and meiosis. One critical function of the kinetochore is to serve as the site of the mitotic spindle attachment checkpoint (SAC), which monitors kinetochore microtubule attachment prior to anaphase onset [1,2]. It is hypothesized that the SAC monitors the attachment of kinetochore microtubules during prometaphase and metaphase, and inhibits anaphase progression until all chromosomes have achieved bipolar spindle attachment. The current model for SAC function suggests that unattached kinetochores recruit checkpoint proteins (such as components of the MAD, BUB, and ZW10/ROD protein complexes), and that these proteins are modified by unattached kinetochores to generate a diffusible signal that delays the onset of anaphase. It has recently been demonstrated that defects in SAC function result in organismal lethality and dominant haploinsufficiency defects. Haploinsufficiency for mouse MAD2, BUB3, or RAE1 results in elevated rates of chromosome missegregation, defects in SAC function, and a predisposition to cancer [3–5], demonstrating the fundamental importance of this checkpoint.

The role of kinetochore localization of SAC components in the generation of the anaphase delay [6] has come into question recently with the published disruption of several inner kinetochore proteins. Disruption of Nuf2 or Ndc80/Hec1 in human cells results in a mitotic arrest, despite the fact that several outer kinetochore components (including Mad2) are unable to sustain kinetochore localization [7,8]. Similarly, disruption of human or chicken CENP-H or CENP-I (the homolog of Schizosaccharomyces pombe Mis6) arrests cells in mitosis for hours, despite the mislocalization of a variety of outer kinetochore proteins, including some SAC components [9–11]. In contrast, disruptions of components of the SAC result in premature entry into anaphase, rather than mitotic arrest or delay. These results suggest that continuous kinetochore localization of SAC components may not be necessary to signal the cell to delay anaphase onset. However, incomplete depletion of the inner kinetochore proteins could produce partially functional kinetochores in which some outer proteins localized properly, while others were mislocalized [8,11,12]. A partially dysfunctional kinetochore could recruit sufficient amounts of SAC proteins to generate an effective checkpoint signal, consistent with a requirement for SAC component recruitment to kinetochores.

A key component of the inner kinetochore is the centromere-specific histone H3-like protein CENP-A [13]. Several recent studies have demonstrated that CENP-A proteins are present in all eukaryotes, and that these proteins are essential for both cell and organismal viability [14–20]. CENP-A proteins replace both copies of histone H3 in centromeric nucleosomes, and physically and genetically interact with all other core histones [14,21–23]. CENP-A proteins are at or near the top of the kinetochore assembly pathway, and are required for the localization of nearly all other kinetochore proteins examined to date, including all tested SAC components [13]. Therefore, cells lacking CENP-A would be expected to contain chromosomes with severely compromised kinetochores, which would be incapable of generating the SAC signal.

Here we report that mutations in the *Drosophila* CENP-A family member (CID, for “Centromere Identifier”) [24] result in an early mitotic delay. Furthermore, *cid* mutants have an intact SAC response to microtubule disruption despite the absence of kinetochore localization of SAC components ROD and BUBR1. We present data that suggest that the DNA damage/repair checkpoint is not responsible for the CID-mediated early mitotic delay. In contrast, the mitotic delay of *cid* mutants is partially suppressed by mutation of the SAC component *bubr1.* We discuss models for the role of SAC proteins in monitoring aspects of kinetochore assembly early in mitosis.

## Results

### CID Null Mutants Display Embryonic Lethality

In a previous study, we found that anti-CID antibody injections into syncitial embryos resulted in phenotypes expected for kinetochore disruption (failure to congress in prometaphase, metaphase arrest, and anaphase segregation defects), but also produced unusual phenotypes (interphase and prophase arrests). However, it was unclear if these phenotypes were the consequence of loss of CID function, an artifact of antibody binding to CID, or a consequence of the specialized nature of the syncitial nuclear divisions. Therefore, we examined the phenotypic consequences of *cid* null mutations in *Drosophila* embryos. The alleles examined were T11–2 (Q51 to stop), T12–1 (Q83 to stop), T21–3 (Q94 to stop), and T22–4 (Q102 to stop) (J. Cecil and T. Kaufman, unpublished observations). All of these alleles are lethal when homozygous, in *trans*-heterozygous combinations, and over a deficiency for the region (unpublished data); thus, the *cid* gene is essential for *Drosophila* development.

**Figure 1:**
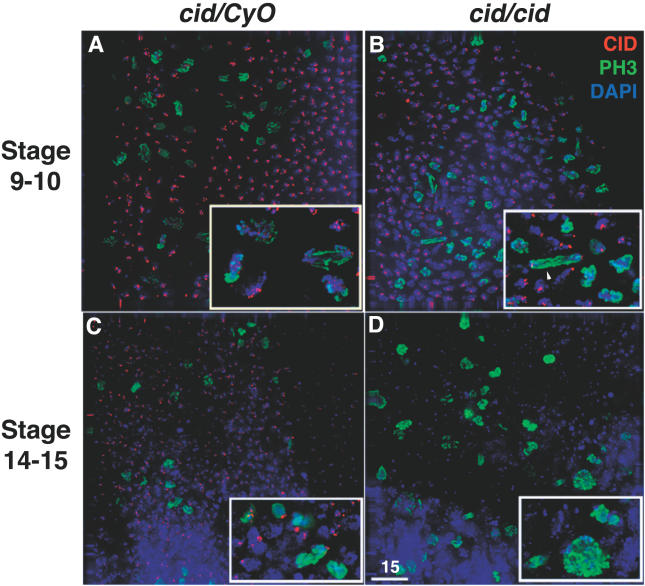
*cid* Null Embryos Exhibit Multiple Mitotic Phenotypes. CID, PH3, and DAPI staining of *cid/CyO* and *cid/cid* embryos at different stages of development were monitored to evaluate mitotic progression and segregation defects. (A) Heterozygous *(cid/CyO)* stage 9–10 embryos displayed no mitotic defects and robust CID staining at kinetochores (inset). (B) *cid* null animals (*trans*-heterozygous for different *cid* alleles, see Materials and Methods) exhibited lagging chromosomes during anaphase. Some CID staining was still visible at this stage, demonstrating that these phenotypes resulted from partial loss of CID function, due to the presence of maternal CID protein. (C) *cid*/*CyO* stage 14–15 embryos show normal mitotic progression and normal CID staining at kinetochores (inset). (D) *cid* null animals exhibited an elevated mitotic index, lower nuclear density, and little detectable CID staining in some cells at stage 14–15. The strong depletion of CID staining suggests that these phenotypes are the result of complete loss of zygotic *cid* function. *cid* null animals have a large number of presumably polyploidy cells (inset) suggesting high levels of aneuploidy due to repeated failures in cell division. Scale bar indicates 15 μm.

To examine the phenotypic consequences of *cid* disruption, crosses were made between parents heterozygous for a *cid* mutation and a balancer that contained an ElaV-LacZ fusion construct, which is expressed in the developing nervous system [25]. We collected embryos from these crosses and stained them for CID, LacZ, histone H3 phosphorylation at serine 10 (PH3) [26], and DNA (DAPI). *cid* null and heterozygous embryos were unambiguously distinguished by the absence or presence (respectively) of ElaV-LacZ expression. *cid* null mutant embryos died around stage 15 of embryogenesis, and displayed a phenotypic series that correlated with the temporal disappearance of maternal CID protein and the absence of newly synthesized zygotic protein. At embryonic stages 9–10, *cid* null embryos displayed lagging chromosomes during anaphase and unresolved chromatin bridges during telophase, which were not observed in heterozygous controls (Figure 1A and 1B). These phenotypes are the result of partial loss of CID protein; staining with anti-CID antibodies demonstrated that maternally derived CID is still present in stage 9–10 embryos, albeit at reduced levels (Figure 1A and 1B). Lagging chromosomes and chromatin bridges are entirely consistent with the phenotypes we observed after partial disruption of CID by RNAi or antibody injection [17,27].

In later stages of development (stages 13–15), high levels of CID staining were observed in heterozygous siblings, whereas in *cid* homozygous mutant embryos, most cells in mitotically active tissues had no visible CID signal (Figure 1C and 1D). The PROD protein binds a satellite DNA near the Chromosomes 2 and 3 centromeres [28], and its localization is not dependent on the presence of CID [17]. Comparison of the levels of CID and PROD staining in homozygous mutant and heterozygous control embryos suggest that approximately 90%–100% of CID was depleted in stage 15 *cid* mutants (Figure S1, see Materials and Methods). Thus, some cells retain small amounts of maternal CID, and these alleles behave as nulls with respect to functional zygotic protein, as predicted from the early stop codons present in the mutations.

Homozygous *cid* null embryos displayed few defects associated with gross morphological patterning or development (unpublished data). However, defects were associated with the organization of the developing nervous tissue, consistent with the fact that few other cell types are actively dividing in later stage embryos, and that the most severe defects are also associated with the nervous tissue in other mitotic mutants that die during embryogenesis [29]. These later stage, terminal embryos displayed a high degree of disorganization of the developing nervous tissue, with obvious micronuclei, large presumably polyploid nuclei, very few true metaphase plates, and few anaphases and telophases. The overall nuclear density was much lower in the *cid* mutants (~1/2 of heterozygous controls), which is also consistent with the aneuploidy that results from failures in chromosome segregation and cell division. These phenotypic characteristics are very similar to a recently described mutation in *Drosophila* CENP-C [30], suggesting that these defects result from disruption of the inner kinetochore.

### CID Disruption Results in an Early Mitotic Delay

**Figure 2:**
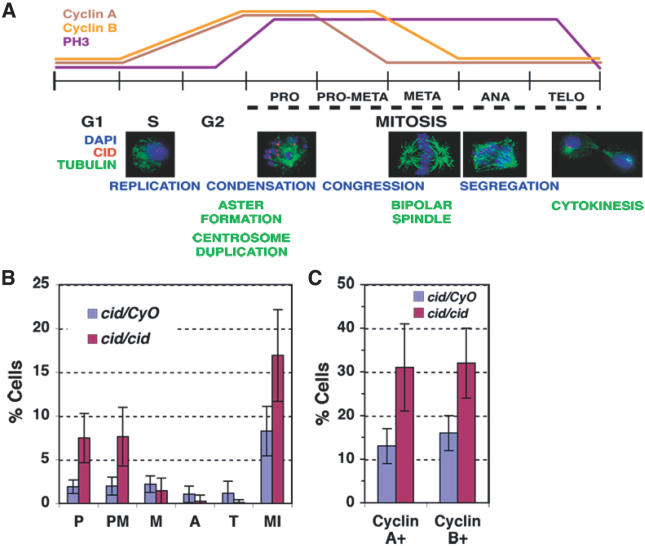
*cid* Null Mutants Exhibit a G2/Prophase Delay. Cell cycle progression was monitored in *cid/CyO* and *cid/cid* embryos by staining for PH3, cyclin A, cyclin B, and tubulin. (A) Schematic diagram of the appearance and destruction of various cell cycle regulatory factors and markers. ANA, anaphase; META, metaphase; PRO, prophase; PRO-META, prometaphase; TELO, telophase. (B) *cid/cid* animals displayed an elevated mitotic index, and an increased number of cells in prophase and prometaphase, compared to *cid/CyO* controls. A, anaphase; M, metaphase; MI, mitotic index; P, prophase; PM, prometaphase; T, telophase. (C) *cid/cid* animals had a 2-fold higher number of cyclin A and B positive cells than *cid/cid* controls. Scale bars indicate 15 μm.

The appearance of H3 phospho-serine 10 (PH3), destruction of the mitotic cyclins, and mitotic spindle morphology can be used to discriminate different stages of G2 and mitosis (Figure 2A). Cyclins A and B begin to accumulate in S and G2 phases [31], and PH3 begins to appear in late G2, and is used as a general marker for mitotic index [32]. Subsequently, cyclin A destruction is observed during prometaphase, cyclin B destruction occurs at the metaphase to anaphase (M:A) transition, and PH3 staining is gradually lost from chromosomes at the end of telophase.

To determine the effects of *cid* depletion on cell cycle progression, the number of cells in specific stages of mitosis was determined by staining homozygous and heterozygous mutant stage 15 embryos for tubulin, cyclin A, cyclin B, and PH3. Three observations demonstrated that *cid* mutants were delayed early in mitosis, predominantly in prophase/prometaphase. First, *cid* mutants displayed a 2.4-fold higher mitotic index (*p* < 0.01) and a 2-fold higher number of cells positive for cyclin A (*p* ≤ 0.01) and cyclin B (*p* ≤ 0.01), in comparison to heterozygous control siblings (Figure 2B and 2C). Second, *cid* mutants showed a marked increase in the number of cells in prophase and prometaphase, as judged by chromosome and spindle morphology (Figure 2B). Third, very few cells progressed to anaphase in *cid* mutants, suggesting that *cid* mutants were delayed prior to the metaphase–anaphase transition (Figure 2B). Thus, complete depletion of CID in embryos results in a mitotic delay, predominantly in prophase and prometaphase.

### Inactivation of the DNA Damage Checkpoint Does Not Abrogate the *cid-*Mediated Mitotic Delay

**Figure 3:**
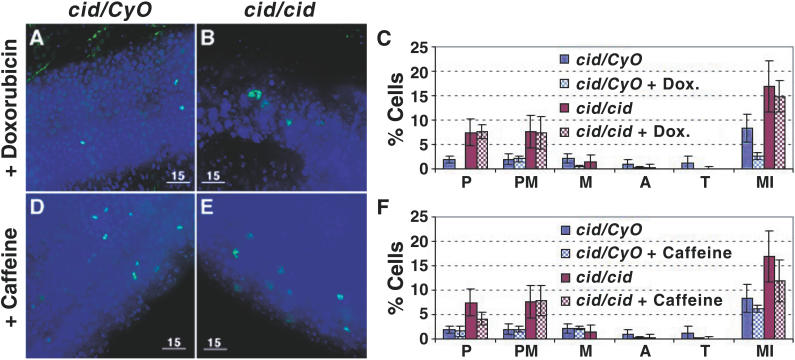
DNA Damage Is Not Responsible for the *cid-*Mediated Mitotic Delay The effect of DNA damage on cell cycle progression was determined by treating stage 15 *cid* null and heterozygous embryos with the topoisomerase II inhibitor doxorubicin. (A–C) *cid/CyO* cells dramatically decreased entry into mitosis in response to DNA damage, whereas *cid*/*cid* cells were unaffected by doxorubicin (Dox.) treatment. (D–F) The MEI-41/ATR kinase was inhibited by treating *cid* homozygous and heterozygous embryos with 2 mM caffeine. Inactivation of the DNA damage checkpoint did not suppress the *cid-*mediated mitotic delay, as the mitotic index of *cid* mutants remained double that of controls, with the majority of the mitotic cells delayed in prophase or prometaphase. A, anaphase; M, metaphase; MI, mitotic index; P, prophase; PM, prometaphase; T, telophase. Scale bars indicate 15 μm.

The mitotic delay observed in homozygous *cid* mutant embryos suggested that *cid* depletion and failure to form a kinetochore activated a cell cycle checkpoint. A recent study in *Xenopus* suggested that DNA damage and repair may be involved in CENP-A assembly at centromeres [33]. Therefore, incomplete kinetochore chromatin assembly or chromosome segregation errors caused by *cid* mutation could result in DNA damage and activation of the DNA damage checkpoint, which would mediate the early mitotic cell cycle delay. To determine whether DNA damage phenocopies the *cid* null mutations, we treated *cid* mutant and heterozygous embryos with doxorubicin, a topoisomerase II inhibitor known to generate dsDNA breaks [34]. We found that doxorubicin treatment dramatically decreased the mitotic index of *cid* heterozygous embryos (Figure 3A–3C), consistent with previous studies of the effects of DNA damage on cell cycle progression [35]. Doxorubicin treatment had little effect on the mitotic index of *cid* homozygous mutant embryos, which likely reflects the fact that these cells were already delayed in mitosis at the time of drug addition. Importantly, the decreased mitotic index in *cid* heterozygotes demonstrates that a DNA damage-induced cell cycle delay results in a fundamentally different (opposite) phenotype from the increased mitotic index observed in untreated *cid* null embryos.

We also performed the reciprocal experiment, to determine if an intact DNA damage checkpoint was necessary for the *cid*-mediated early mitotic delay. A central component of the DNA damage response, the MEI-41 ATR kinase [35], was inhibited by treating *cid* mutant and heterozygous embryos with 2 mM caffeine [34]. We found that caffeine treatment of *Drosophila* embryos phenocopied *mei-41* and *grapes* maternal affect mutations, and is likely to completely inactivate the DNA damage checkpoint (unpublished data). Inactivation of MEI-41 by caffeine treatment did not suppress the *cid-*mediated mitotic delay. The mitotic index of *cid* mutants remained nearly twice that of heterozygous controls, and most of the mitotic cells were found in prophase or prometaphase, with very few cells progressing to later stages of mitosis (Figure 3D–3F). These results demonstrate that inactivation of the DNA damage checkpoint does not abrogate the *cid*-mediated mitotic delay, and confirms that this delay is not the result of DNA damage induced by *cid* mutations.

### *cid* Mutant Cells Have an Intact SAC Response to Microtubule Disruption

The other major cell cycle checkpoint that could be responsible for the cell cycle delay observed in *cid* mutants is the SAC, which monitors kinetochore microtubule attachment and regulates the metaphase to anaphase transition. However, the *cid*-mediated mitotic delay appears temporally and phenotypically distinct from SAC-mediated cell cycle effects. When the SAC is activated (e.g., by the addition of microtubule polymerization inhibitors), cyclin A is degraded, but not cyclin B [31,36] (compare to Figure 2), and cells arrest in prometaphase/metaphase with condensed but unaligned chromosomes. Finally, the absence of normal kinetochore formation in all cases in which CENP-A proteins have been depleted or mutated [15–20,37] suggests that *cid* null mutant cells should not have an intact SAC, and that SAC components should not play a role in the *cid*-mediated mitotic delay.

**Figure 4:**
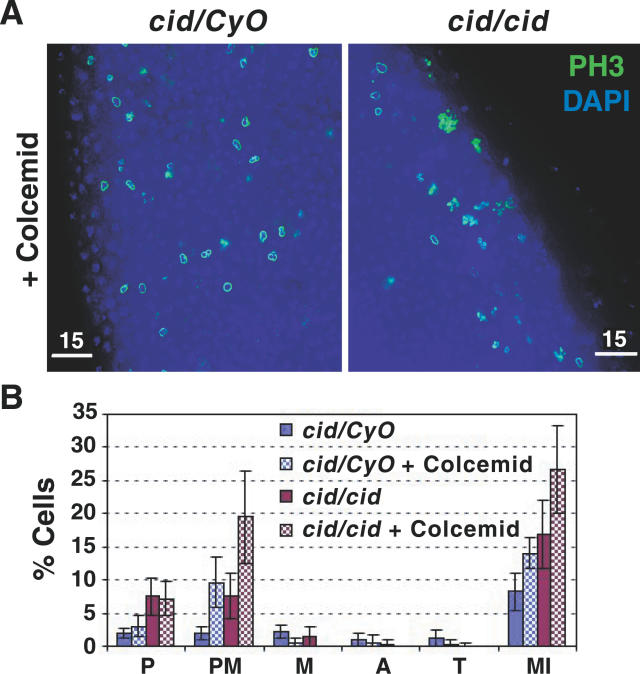
*cid* Mutants Retain an Intact SAC Response to Microtubule Depolymerization *cid* null and heterozygous embryos were treated with colcemid to determine if they have an intact SAC response to spindle disruption. Both *cid/CyO* and *cid/cid* cells were able to delay the cell cycle in response to spindle disruption (A), as evidenced by an approximately 2-fold increase in mitotic index and an accumulation of cells in prometaphase (B). A, anaphase; M, metaphase; MI, mitotic index; P, prophase; PM, prometaphase; T, telophase. Scale bars indicate 15 μm.

To directly test for the presence of an SAC response to microtubule disruption in *cid* mutant animals, we treated stage 15 *cid* homozygous and heterozygous animals with the microtubule depolymerizing agent colcemid. We found that both homozygous and heterozygous cells were delayed in response to colcemid treatment (Figure 4), consistent with the observation that the Saccharomyces cerevisiae CENP-A homolog Cse4 is not required for SAC function [38]. First, both genotypes displayed a nearly 2-fold increase in mitotic index after 1 h of treatment (Figure 4B). Second, the increased mitotic index was accompanied by a large increase in the number of cells accumulated in prometaphase in both *cid* null and heterozygous animals. We conclude that *cid* mutant cells retain an intact SAC response to microtubule disruption; thus, SAC components could play a role in the *cid*-mediated early mitotic delay. In addition, the fact that some *cid*/*cid* cells accumulated in prometaphase/metaphase after colcemid treatment indicates that cells can eventually overcome the prophase delay, and that prometaphase is likely to be the terminal arrest point, similar to an SAC-mediated cell cycle arrest.

### Mutation of the SAC Component *bubr1* Partially Suppresses the *cid-*Mediated Mitotic Delay

**Figure 5:**
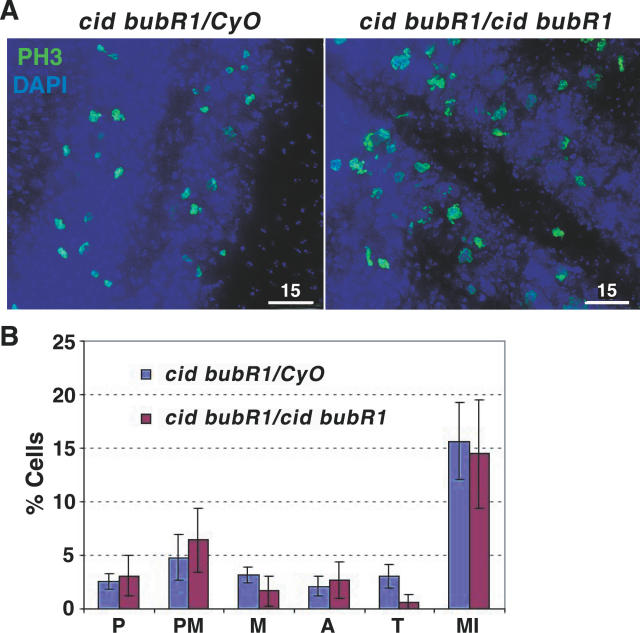
A *bubr1* Mutation Partially Suppresses the *cid*-Mediated Mitotic Delay (A) *cid bubr1* double mutants were examined for mitotic progression by staining for PH3 and DAPI. *cid bubr1* mutants show an increased nuclear density and number of anaphases compared to *cid* single mutants. Scale bar indicates 15 μm. (B) *bubr1* suppressed the high mitotic index and high number of cells delayed in prophase and prometaphase observed in *cid* single mutants (compare to ratios in Figure 2B). A, anaphase; M, metaphase; MI, mitotic index; P, prophase; PM, prometaphase; T, telophase.

To directly examine the role of the SAC in the *cid*-mediated mitotic delay, we determined if a mutation that inactivates the SAC can restore normal cell cycle progression. *cid bubr1* double mutants were generated, and homozygous and heterozygous double mutant embryos were monitored for cell cycle progression by staining for PH3. Surprisingly, *bubr1* mutations partially suppressed most of the cell cycle phenotypes associated with *cid* mutation (Figure 5A and 5B). The mitotic index in *cid bubr1* double mutants was nearly the same as observed in heterozygous controls (1.1-fold, *p* = 0.6, *cid bubr1/cid bubr1* compared to *cid bubr1/CyO*), compared to the 2.4-fold increase observed for *cid/cid* mutants over controls (see above). The number of cells delayed in prophase and prometaphase also decreased dramatically in *cid bubr1* double mutants and was comparable to heterozygous controls, whereas the number of cells in anaphase showed a corresponding increase and was greater that controls. Note that *cid bubr1* double mutants had a mitotic index nearly double that of *cid* single mutants, for reasons that are unclear at this time. We eliminated bias that could arise from this difference by only comparing homozygous double mutants to heterozygous double mutants (see Materials and Methods for a more detailed discussion). We conclude that inactivation of a component of the SAC relieves the *cid*-mediated mitotic delay, suggesting that at least one component of the SAC is involved in delaying cell cycle progression in the absence of CID.

### CENP-C and the SAC Components BUBR1 and ROD Are Unable to Localize to Kinetochores in *cid* Mutants

**Figure 6:**
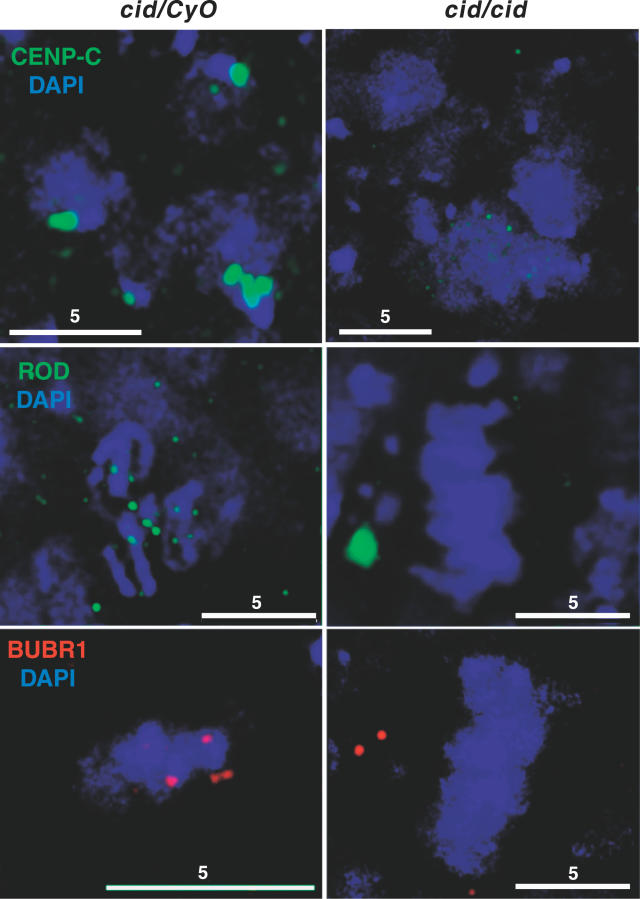
Inner and Outer Kinetochore Protein Localizations Are Disrupted in *cid* Mutant Embryos Kinetochore localization of CENP-C, ROD, and BUBR1 were determined in stage 15 embryos. In *cid/CyO* control embryos (left), all three proteins were localized to the centromere/kinetochore during interphase (CENP-C) or the early stages of mitosis (ROD and BUBR1). All three proteins were absent from centromeres/kinetochores in *cid/cid* animals (right); in some cases, CENP-C was mislocalized in a diffuse pattern. Scale bars indicate 5 μm.

It has been proposed that the APC (anaphase-promoting complex) inhibitory signal is generated by the rapid turnover of SAC proteins at unattached kinetochores [39–43]. We previously demonstrated that all tested outer kinetochore proteins (ROD, BUBR1, Cenp-meta, and POLO) fail to localize to kinetochores in CID-depleted tissue culture cells and CID antibody-injected embryos [17]. CENP-A disruptions in *Caenorhabditis elegans,* mouse, and human cells also result in failure to properly localize kinetochore components, including SAC proteins [15,17–19,37]. Disruption of kinetochore formation and SAC protein localization in *cid/cid* embryos was determined by staining for inner and outer kinetochore proteins. We found that the inner kinetochore protein CENP-C [30] was absent in most *cid/cid* cells, and occasionally was mislocalized in a diffuse pattern throughout the cell, consistent with studies in other organisms and with a severe disruption of kinetochore assembly (Figure 6). Consistent with these results, the SAC components ROD and BUBR1 were unable to localize to kinetochores in stage 15 *cid* null animals, whereas BUBR1 and ROD were localized to kinetochores during all stages of mitosis in heterozygous controls (Figure 6). We conclude that *cid* null mutations delay cells in early mitosis in the absence of sustained kinetochore localization of essential components of the SAC, despite the requirement for at least one of these SAC components (BUBR1).

## Discussion

We have shown that null mutations in the *Drosophila* member of the CENP-A protein family result in embryonic lethality after depletion of maternal CID protein. CID-depleted embryonic cells display an early mitotic delay, consistent with cell cycle defects observed after CID antibody injection [17], suggesting the involvement and activation of a cell cycle checkpoint. This result is similar to a recent knockout of CENP-A in chicken DT-40 cells and a CENP-C mutation in *Drosophila,* both of which resulted in a mitotic delay in the absence of kinetochore assembly [20,30]. However, these studies did not determine when the delay occurred in mitosis, whether the mitotic delays involved the SAC or the DNA repair checkpoint, or whether similar responses to CENP-A depletion occurred in animals.

We addressed the possible involvement of two known cell cycle checkpoints in the CID-mediated early mitotic delay, specifically the DNA damage and SACs. A recent study suggested that DNA damage and repair may be involved in CENP-A assembly in *Xenopus* [33], raising the possibility that elimination of CID alters centromeric chromatin, resulting in DNA damage at the centromere. We addressed this hypothesis in two complementary ways. First, we compared the *cid* mutant mitotic delay phenotypes to the behavior of cells after inducing general DNA damage with doxorubicin. Induction of DNA damage resulted in a reduced mitotic index, not the increased mitotic index observed in the *cid* mutant embryos. Second, we disrupted the DNA damage checkpoint in *cid* mutant embryos using caffeine treatment, which inhibits MEI-41 (ATR), an essential component of the DNA damage response [34,35]. Caffeine treatment did not abrogate the *cid*-mediated mitotic delay. We conclude that DNA damage does not appear to be the signal that induces the *cid*-mediated early mitotic delay, and that this delay does not require an intact DNA damage checkpoint.

These results led us to address the possible involvement of the SAC in the CID-mediated early mitotic delay in animals. The SAC monitors microtubule attachments to the kinetochore; if normal bipolar attachments are not formed, activation of the SAC blocks entry into anaphase, resulting in a prometaphase/metaphase arrest [34,35]. The fact that the CID-mediated delay occurred earlier in mitosis than expected for activation of the SAC suggested that this checkpoint might not be involved. However, we observed that *cid* null mutant cells retained an intact SAC response to microtubule disruption by colcemid, which is similar to the response of Cse4 mutants in *Sa. cerevisiae* [38]. In addition, mutating an essential SAC component (BUBR1) resulted in abrogation of the CID-mediated delay. Previous studies suggested that kinetochore localization of SAC proteins (e.g. MAD2, BUBR1, ROD, and CENP-E) is absolutely required for SAC function. Nevertheless, we observed that BUBR1 and ROD, and the inner kinetochore protein CENP-C, lacked kinetochore localization in *cid* mutant embryos. These results suggest that the CID-mediated early mitotic delay involves the SAC, and that BUBR1 is serving a kinetochore-independent role in delaying mitotic progression, as suggested by recent studies in human and yeast cells [12,44,45].

### Why Do *cid* Mutants Display an Early Mitotic Delay That Is BUBR1-Dependent?

Based on the previous observation of interphase/prophase arrest after CID antibody injection into embryos, we proposed that cells monitor kinetochore assembly early in mitosis, in addition to monitoring the presence of bipolar attachments later in mitosis [17]. It is also possible that *cid* null kinetochores may be able to recruit normal levels of SAC components at early stages of mitosis, but are unable to retain functional levels later in mitosis, as observed for disruption of human Hec1 and Nuf2 and DT-40 CENP-A [7,20].

Alternatively, mitotic arrest may occur in the absence of kinetochore localization of SAC components. Consistent with this hypothesis, the *cid-*mediated early mitotic delay requires at least one SAC component (BUBR1), yet occurs without sustained kinetochore localization of multiple, essential components of the SAC (reported here and in [17]). The finding that defects in kinetochore assembly lead to a BUBR1-dependent early mitotic delay is supported by several recent studies. Disruption of chicken CENP-A, CENP-H, or CENP-I, all inner kinetochore proteins, delays cells in mitosis for hours [9–11,20]. These results suggest that the SAC is able to respond to multiple types of signals and inhibit cell cycle progression.

**Figure 7:**
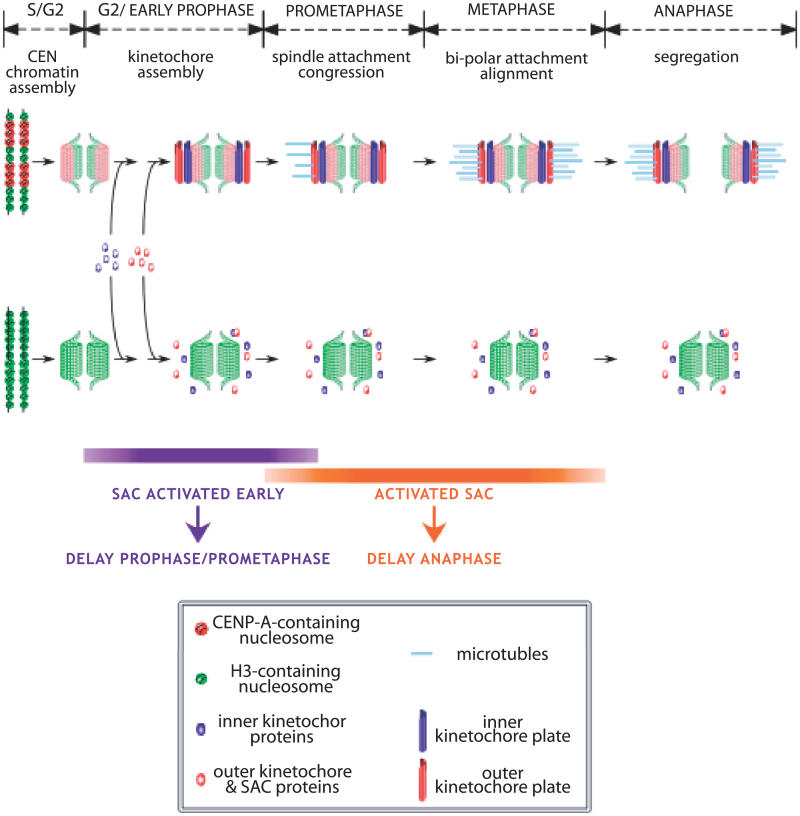
SAC Components Affect Cell Cycle Progression in the Absence of Kinetochore Localization In normal cells, CENP-A chromatin assembly is followed by the recruitment of inner and outer kinetochore proteins [17]. We propose that until kinetochore assembly is complete, free SAC components may be responsible for cell cycle inhibition (early activation of the SAC). Upon completion of kinetochore assembly, SAC components delay anaphase until all chromosomes have achieved bipolar spindle attachment. In *cid* null mutants, both inner and outer kinetochore proteins are free, resulting in a SAC-dependent early mitotic delay that does not depend on localization of SAC components to kinetochores.

How could SAC components contribute to cell cycle delay early in mitosis, prior to their well-established role in monitoring bipolar attachments in prometaphase/metaphase? Loss of CENP-A proteins blocks kinetochore assembly, which may generate “free” (non-kinetochore localized) SAC complexes capable of inhibiting mitotic progression (Figure 7). Since the active inhibitory complex for the SAC is present throughout the cell cycle [46], the complete absence of kinetochore assembly, or the presence of “free” SAC components, could block cells early in mitosis by chronically activating the SAC. It has recently been shown that both BUBR1 and MAD2 function in a kinetochore-independent manner to regulate the length of mitosis, in addition to monitoring kinetochore-microtubule attachments [12,45]. Furthermore, recent studies in *Drosophila* have revealed a role for Bub3 in G2 and early mitosis in promoting the accumulation of mitotic cyclins [47], suggesting that components can ensure normal mitotic progression by inhibiting the APC in a kinetochore-independent manner. This interpretation is also consistent with recent studies that demonstrate that SAC proteins play multiple roles in cell cycle regulation [48–50]. For example, mutations in *Drosophila bubr1* have been shown to bypass the SAC, and are also able to suppress mutations that activate both the DNA damage and SAC in early embryos [48,51]. Furthermore, it has recently been demonstrated that SAC components are responsible for mediating a mitotic arrest in response to DNA damage in vertebrate cells [34], and the mitotic arrest in response to spindle malorientation in *Sc. pombe* [52]. These results strengthen the conclusion that the SAC can respond to more than bipolar kinetochore microtubule attachment, and suggest multiple roles for SAC components in cell cycle regulation. Therefore, the most likely explanation for the *cid-*mediated mitotic delay is that inhibitory SAC complexes can be formed in the absence of kinetochore localization (Figure 7).

The role of the kinetochore in cell cycle progression and the functions of SAC components are clearly more complex than previously thought. Future studies should focus on identifying the components and mechanisms responsible for the *cid-*mediated mitotic delay, and determining if this complex is identical to the standard SAC inhibitory complex.

## Materials and Methods

### Cytology.

*cid* mutant embryos were collected from interallelic crosses and stained as described using either a formaldehyde or MeOH:EGTA fixation. *Trans*-heterozygous combinations of the different *cid* alleles were generated in order to eliminate phenotypic effects of other lethal mutations present on each of the *cid* mutant chromosomes (unpublished data). All of the data presented were obtained for crosses between *cid^11–2^* and *cid^22–4^*, although crosses between other alleles produced identical phenotypes. Antibodies used were cyclin A [31], cyclin B [31], LacZ (Sigma, St. Louis, Missouri, United States), tubulin (Sigma), ROD [53], BUBR1 [51], and CID [17]. For quantitation of mitotic index and cyclin abundance, all cells within the developing central nervous system were counted from at least five mutant and five control embryos. The ratios presented are the number of PH3- or cyclin-positive cells divided by total cells, in order to normalize for the lower nuclear density present in *cid* mutant embryos. Quantification of the stages of mitosis was performed by costaining embryos for PH3 and tubulin. The distinction between prophase and prometaphase was made as follows: Prophase was classified as chromosomes with incomplete condensation (i.e., round PH3+ nucleus), in which no individual chromosomes or chromosome arms were visible and DNA was not obviously aligning at the metaphase plate. Prophase tubulin staining showed bright centrosomal signals with little or no obvious microtubules interacting with the chromosomes. Prometaphase was classified as chromosomes with complete condensation (i.e., clearly visible individual chromosomes and chromosome arms) in which the chromosomes were clearly in the process of aligning at the metaphase plate. Tubulin staining showed a focused bipolar microtubule array that was clearly interacting with the chromosomes.

For quantification of CID levels in mitotically active cells in mutant and control embryos (Figure S1), the sum of pixel values for both CID and PROD immunofluorescence from five to seven embryos was obtained using the two-dimensional polygon finding tool in softWoRx (Applied Precision, Issaquah, Washington, United States). The pixel values were summed and presented as a ratio of CID:PROD, to provide a rough estimate of the amount of CID depletion in each embryo. Based on these ratios, 90%–100% of CID was depleted in stage 15 *cid/cid* mutants, relative to heterozygous controls, suggesting retention of a small amount of maternal protein in some cells. For all quantitations, standard deviations were calculated per embryo, and data were compared using the Student *t* test. Note that the amount of CID depletion in *cid* homozygotes is likely to be an underestimate (up to 2-fold) with respect to wild-type embryos, since *cid* mutant heterozygotes were used as the quantitation controls.

All images were acquired using a DeltaVision workstation (Applied Precision) and analyzed using softWoRx software, as described previously [17].

### Drug treatments.

*cid* mutant and heterozygous embryos were bleach dechorionated and incubated in a 1:1 mixture of Schneider's medium (+10% heat-inactivated FBS) and octane as described in [54]. Colcemid was used at a concentration of 3 μg/ml for 1 h, caffeine was used at a concentration of 2 mM for 2 h, and doxorubicin was used at a concentration of 2 μM for 2 h. After drug treatment, embryos were fixed using formaldehyde and processed for immunofluorescence as described above.

### Genetics.

The *bubr1* allele used was k03113, and was obtained from the Bloomington Stock Center (Bloomington, Indiana, United States). *cid bubr1* double mutants were generated by recombination using standard methods.

Mutations in *cid* (*centromere identifier*/CG13329) were recovered in genetic screens designed to isolate new mutant alleles of *cnn (centrosomin)* [55]. The *cid* locus is tightly linked to *cnn* in the 50A region of the right arm of the second chromosome in D. melanogaster. The genes in this genomic region, proximal to distal, are *cnn* (*centrosomin*/CG4832), *Cbs* (*centrosomin's beautiful sister*/CG4840), *arr* (*arrow*/CG5912), *cbc* (*crowded by cid*/CG5970), *cid* (*centromere identifier*/CG13329), *bbc* (*b-b in a boxcar*/CG6016), and *drk* (*downstream of receptor kinase/*CG6033). The initial characterization of this region included screening of cDNA libraries and expressed sequence tag (EST) collections to produce transcript profiles for each of these genes, and saturation mutagenesis screens to recover recessive lethal and sterile mutations. Breakpoint-associated mutations, principally deletions, and complementation analyses were used to localize each of the newly recovered mutations to the individual molecularly defined and computationally identified transcription units. Using primers designed from genomic and cDNA sequences the mutant alleles of each locus were sequenced and the genetic localization of the complementation groups confirmed. This screen resulted in the recovery of the four alleles of *cid* reported in this paper: *cid^t11–2^* Q51 to stop, *cid^t12–1^* Q83 to stop, *cid^t21–3^* Q94 to stop, and *cid^t22–4^* Q102 to stop.

### Examination of *cid bubr1* heterozygous mutants.

During the course of scoring the mitotic parameters of *cid bubr1* double mutants, we noticed that *cidbubr1* single mutants had a mitotic index nearly twice as high as *cid* single mutants alone, which prompted us to investigate these heterozygous mutants further for possible haploinsufficiency effects. We found no incidence of chromosome segregation defects in *cid bubr1/CyO* embryos despite their elevated mitotic index. We also examined mitotic tissue of *cid bubr1/CyO* third instar larval brains because this tissue allows a more precise karyotypic analysis and could reveal subtle defects not seen in embryonic tissue. We found that *cid bubr1/CyO* animals had a higher mitotic index than *cid/CyO* animals (1.10 [*n* = 315 fields] vs. 0.76 [*n* = 400 fields]), yet we did not find any evidence for aneuploidy or mitotic defects in any of the mitotic figures examined. We then determined whether *cid bubr1* and *cid* heterozygous animals had a normal response to colcemid treatment by incubating brains with colcemid for 1 h. We found that *cid bubr1* and *cid* heterozygous mutants had a normal response to colcemid treatment (*cid/CyO* mitotic index increased from 0.86 to 2.17 [*n* = 551 fields], and *cid bubr1/CyO* mitotic index increased from 1.10 to 2.37 [*n* = 264 fields]). From this data we conclude that *cid* and *cid bubr1* mutants do not have a haploinsufficient effect on mitosis, and that there are likely to be other factors in the genetic background that lead to the increased mitotic index of *cid bubr1* double mutants. To avoid interpretation artifacts that might be caused by this difference, in all cases we only compared data from *cid bubr1* homozygotes to *cid bubr1* heterozygotes, and *cid* homozygotes to *cid* heterozygotes.

## Supporting Information

Figure S1 Quantification of the Amount of CID Depletion in *cid* Mutant Embryos Stage 15 *cid/cid* and *cid/CyO* embryos were stained for CID and PROD. PROD was present in a punctate pattern in both genotypes. Estimation of the amount of CID depletion was preformed by comparing the ratio of total CID staining to PROD staining in five different mutant and heterozygous embryos. From this analysis we estimate that 90%–100% of CID protein is depleted in mutant embryos.
(4.0 MB PDF)

## Abbreviations

SAC: spindle attachment checkpoint
