# Supplementary material for: Drosophila CENP-A Mutations Cause a BubR1- Dependent Early Mitotic Delay without Normal Localization of Kinetochore Components
Source: PLoS Genet. 2006 Jul 14;2(7):e110. doi: 10.1371/journal.pgen.0020110 (PMC1500813; doi:10.1371/journal.pgen.0020110)

Figure S1

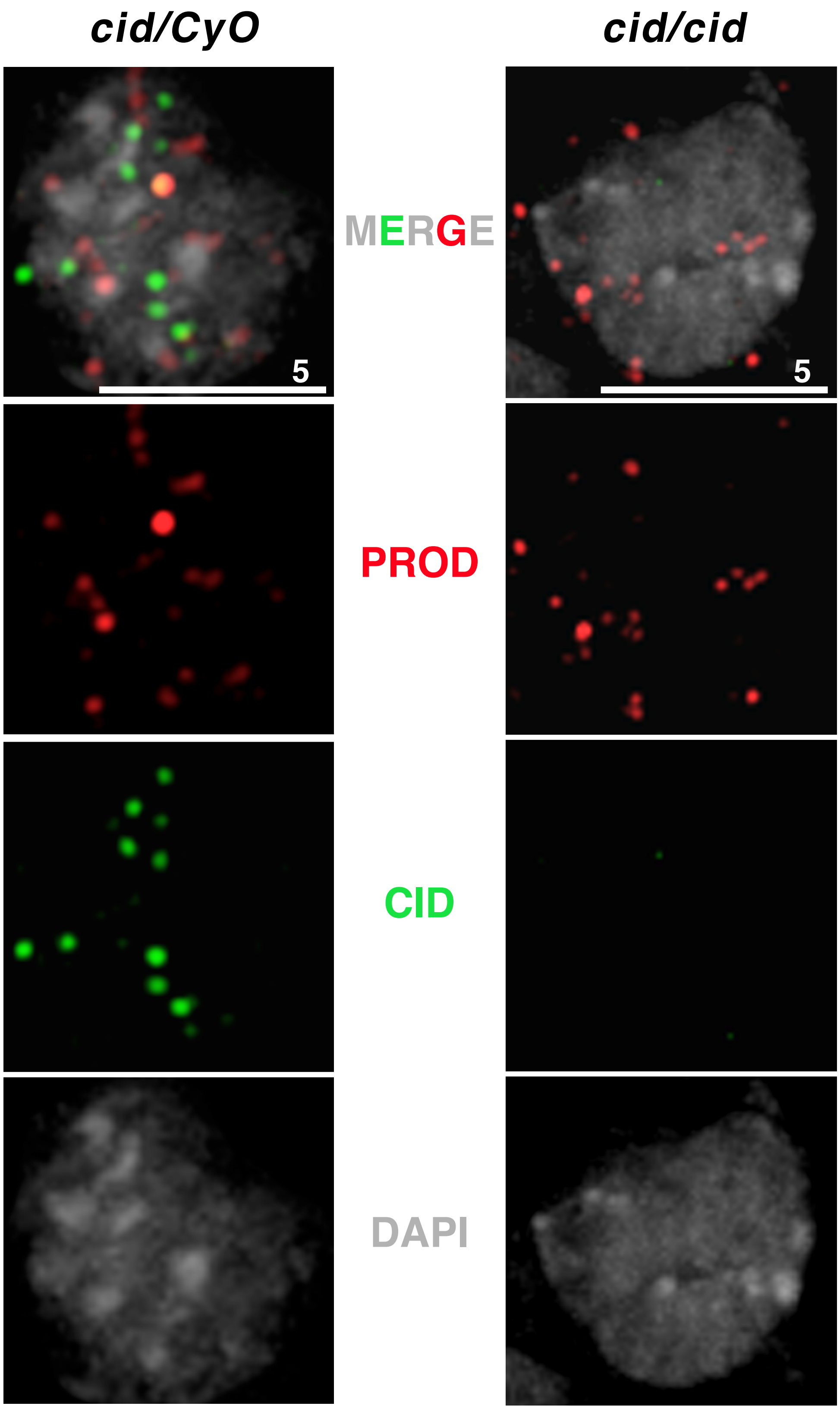

Supplement: Supplementary file 1 [file pgen.0020110.sg001.pdf]
